# Loss of maternal ANNEXIN A10 via a 34-kb deleted-type copy number variation is associated with embryonic mortality in Japanese Black cattle

**DOI:** 10.1186/s12864-016-3312-z

**Published:** 2016-11-24

**Authors:** Shinji Sasaki, Takayuki Ibi, Takayuki Akiyama, Moriyuki Fukushima, Yoshikazu Sugimoto

**Affiliations:** 1 Shirakawa Institute of Animal Genetics, Japan Livestock Technology Association, Odakura, Nishigo, Fukushima 961-8061 Japan; 2 Graduate School of Environmental and Life Science, Okayama University, Tsushima-naka, Okayama 700-8530 Japan; 3 Northern Center of Agricultural Technology, General Technological Center of Hyogo Prefecture for Agriculture, Forest and Fishery, Asago, Hyogo Japan

**Keywords:** Embryonic mortality, Copy number variation, Threshold model, ANNEXIN A10, Maternal effect, Beef cattle

## Abstract

**Background:**

Conception is a fundamental trait for successful cattle reproduction. However, conception rates in Japanese Black cattle have been gradually declining over the last two decades. Although conception failures are mainly caused by embryonic mortality, the role of maternal genetic factors in the process remains unknown. Copy number variation (CNV), defined as large-scale genomic structural variants, contributes to several genetic disorders. To identify CNV associated with embryonic mortality in Japanese Black cattle, we evaluated embryonic mortality as a categorical trait with a threshold model and conducted a genome-wide CNV association study for embryonic mortality using 791 animals.

**Results:**

We identified a deleted-type CNV ranging from 378,127 to 412,061 bp on bovine chromosome 8, which was associated with embryonic mortality at 30–60 days after artificial insemination (AI). The CNV harbors exon 2 to 6 of ANNEXIN A10 *(ANXA10)*. Analysis of sequence traces from the CNV identified that 63 bp reads bridging the breakpoint were present on both sides of the CNV, indicating that the CNV was generated by non-allelic homologous recombination using the 63 bp homologous sequences. Western blot analysis showed that the CNV results in a null allele of *ANXA10*. This association was replicated using a sample population size of 2552 animals. To elucidate the function of ANXA10 in vivo, we generated *Anxa10* null mice using the CRISPR/Cas9 system. Crossbreeding experiments showed that litter size from crosses of both *Anxa10* ^-/-^ and *Anxa10* ^+/-^ females had fewer pups than did *Anxa10* ^+/+^ females, and embryos of *Anxa10* ^-/-^ females died between implantation stages E4.5 and E12.5. These results indicate that loss of maternal *Anxa10* causes embryonic mortality.

**Conclusions:**

This study identified a deleted-type CNV encompassing *ANXA10* in cows that was associated with embryonic mortality at 30–60 days after AI. Using a mouse model, we confirmed that litter sizes were smaller in crosses of both *Anxa10* ^-/-^ and *Anxa10* ^+/-^ females relative to those of wild females. These results indicate that *ANXA10* is a maternal factor that is critical for embryo development.

**Electronic supplementary material:**

The online version of this article (doi:10.1186/s12864-016-3312-z) contains supplementary material, which is available to authorized users.

## Background

**Fig. 1:**
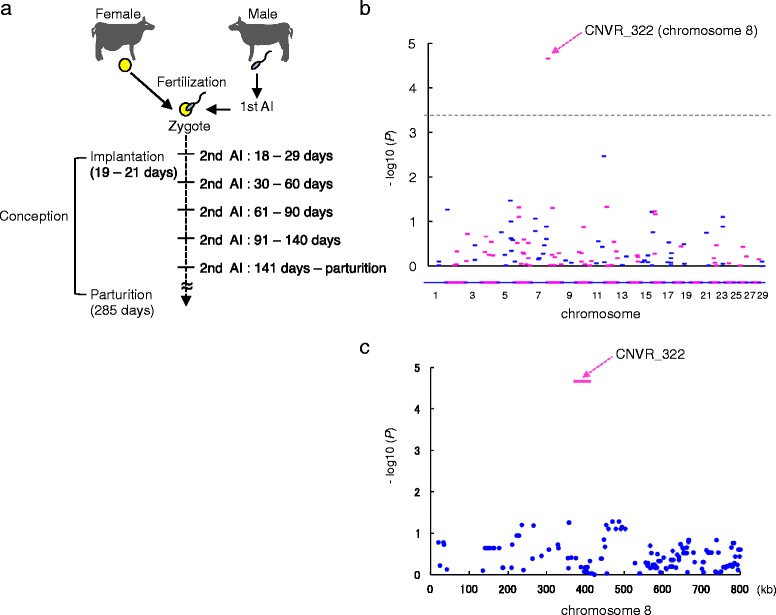
The association of CNVRs with embryonic mortality in 791 Japanese Black cattle. **a** Schematic representation of artificial insemination (AI) breeding program in Japanese Black cattle. At each parity, female cattle were artificially inseminated (1st AI) at the onset of estrus. Second AI (2nd AI) was carried out after the cow showed estrus. The resumption of estrus after the 1st AI indicated that the fertilization or embryo development derived from the 1st AI had failed. Thus, embryonic mortality was defined by cows that received a 2nd AI at 18–29 days (D), 30–60 D, 61–90 D, 91–140 D, and 141 D–parturition after the 1st AI. **b** Manhattan plot of the association of 116 deleted-type CNVRs with embryonic mortality at 30–60 D in 791 Japanese Black cattle. Chromosomes are distinguished with alternating colors (*blue*, odd numbers; *red*, even numbers). Dashed line is the Bonferroni-corrected threshold for genome-wide significance (-log10 (*P*) = 3.365). **c** Manhattan plot of the association of the surrounding SNPs, which are derived from Illumina BovineHD BeadChip array, at approximately ± 400 kb distance from CNVR_322. Positions are based on the UMD3.1 assembly of the bovine genome

Over the past two decades, conception rates for artificial insemination (AI) breeding programs in Japanese Black cattle have been gradually declining (e.g., first-AI conception rates have decreased from 67.4 to 56% between 1992 and 2012 in Japan) [1]. This trend has also been observed in dairy cattle [1, 2]. In contrast, the fertilization rates for a single natural service in cattle have been reported to range from 88 to 90% in various breeds, reviewed in [3]. The difference between “conception rate” and “fertilization rate” is approximately 35%, suggesting that embryonic mortality occurs mainly after postzygotic development, reviewed in [3, 4] (Fig. 1a). Therefore, researchers have aimed to identify the genetic factors involved in embryonic mortality during postzygotic development to improve reproductive performance and profitability in cattle.

Copy number variation (CNV) is defined as deleted or duplicated segments of genome that range from 1 kb to several Mb, reviewed in [5]. CNVs cause diseases via various mechanisms such as gene dosage modification and gene structure disturbance, either directly by exposing recessive alleles or by disturbing the regulatory regions of genes, reviewed in [6]. Recently, two studies of Holstein and Nordic Red cattle found that deleted-type CNVs were associated with infertility [7, 8]. More specifically, the associated regions harboring the causative CNVs were identified using SNP array, as these CNVs were in linkage disequilibrium (LD) with their neighboring SNPs. However, genome-wide CNV screening for embryonic mortality has not been directly applied to cattle populations. Recently, we reported genome-wide CNV maps for 1481 Japanese Black cattle, identified using the Illumina Bovine High-Density (HD) BeadChip Array [735,293 single-nucleotide polymorphisms (SNPs), with an average marker interval of 3.4 kb on the autosomes] [9]. These high-resolution CNV maps enabled us to directly scan the CNVs associated with embryonic mortality in Japanese Black cattle.

In this study, we evaluated embryonic mortality as a categorical trait with a threshold model [10, 11] and conducted a genome-wide CNV association study using the traits. We identified a deleted-type CNV, encompassing ANNEXIN A10 (*ANXA10*), which was associated with embryonic mortality at 30–60 days after AI.

## Results and discussion

### Embryonic mortality was evaluated as a categorical trait with a threshold model in Japanese Black cattle

Embryonic mortality was defined in cows that received a second round of AI (2nd AI) at 18–29 days (D), 30–60 D, 61–90 D, 91–140 D, and 141 D–parturition after the first AI (1st AI) (Fig. 1a). The 2nd AI was performed when estrus resumed after the 1st AI in our AI breeding program. The resuming of estrus after the 1st AI indicated that fertilization or conception derived from the 1st AI had failed. Embryonic mortality was estimated from 785,993 mating records, derived from 79,617 reproductive females. The embryonic mortality rate in each period is shown in Table 1. As cattle are a mono-ovulatory species and usually delivers a single calf at each parity, the embryonic mortality in cattle was naturally expressed as a binary categorical trait. Thus, we estimated genetic parameters in a threshold model using Bayesian analysis with Gibbs sampling. The threshold model assumes an unobservable underlying continuous variable (liability), with a threshold deciding the observable binary outcome [11–13]. Table 2 shows the estimates of variance components and genetic parameters for embryonic mortality for each period. The estimates of direct heritability of embryonic mortality at each stage were estimated to be 0.01–0.03, consistent with previous reports of female fertility and conception-related traits in cattle, reviewed in [14, 15].

**Table 1:** Number of records and embryonic mortality rate (%) at each period

2nd AI	Number of records	Embryonic mortality rate (%)
18–29 D	769,103	23.00
30–60 D	592,243	21.97
61–90 D	462,098	16.91
91–140 D	383,979	8.47
141 D–parturition	351,455	3.30

Embryonic mortality was defined by cows that received a second round of AI at 18–29 days (D), 30–60 D, 61–90 D, 91–140 D, and 141 D–parturition after the 1st AI

**Table 2:** Posterior means of variance components and genetic parameters for embryonic mortality using the threshold model

2nd AI	σ_a_^2^	σ_pe_^2^	σ_e_^2^	σ_p_^2^	h_a_^2^	c^2^	R
18–29 D	0.02	0.04	1.00	1.05	0.01	0.04	0.05
30–60 D	0.01	0.03	1.00	1.04	0.01	0.02	0.03
61–90 D	0.03	0.07	1.00	1.10	0.03	0.06	0.09
91–140 D	0.02	0.09	1.00	1.11	0.02	0.08	0.10
141 D–parturition	0.01	0.08	1.00	1.09	0.01	0.07	0.09

σ_a_^2^, direct genetic variance; σ_pe_^2^, permanent enviromental variance; σ_e_^2^, residual variance; σ_p_^2^, phenotypic variance; h_a_^2^, direct heritability; c^2^, proportion of phenotypic variance due to permanent enviromental varinace; R, repeatablitity

### CNVR_322 was associated with embryonic mortality on chromosome 8 in Japanese Black cattle

We previously identified 861 CNV regions (CNVRs), defined as the union region of overlapping CNVs detected in at least two animals [16], in the autosomes of 1481 Japanese Black cattle [9]. Of the 1481 animals, 791 cows were evaluated for genetic parameters of embryonic mortality as described above. Several studies have demonstrated that deleted chromosome segments, deleted-type CNVs, are directly connected to diseases in cattle [8, 17–20]; thus, we evaluated the association between deleted-type CNVRs and embryonic mortality in this study. Of 861 CNVRs, we selected 116 of the deleted-type CNVRs that were present at a frequency of > 1% in 1481 animals [9] (Additional file 1: Table S1). We conducted a genome-wide CNV association study with the breeding value of embryonic mortality in each period using GEMMA software [21]. The genomic inflation factor (λGC) in this analysis was 1.012, indicating that the samples were appropriate for an association study. We found that one CNVR, CNVR_322, was associated with embryonic mortality at 30–60 D after AI, reaching the Bonferroni-corrected threshold (*P* < 4.31 × 10 ^−4^, Fig. 1b); however, it was not associated with embryonic mortality in other periods or overall conception rate (Additional file 1: Table S2). The CNVR_322 was located within a 30,865 bp window from 380,524 bp to 411,389 bp on bovine chromosome 8 (*P* = 2.19 × 10^−5^) (Table 3). Of note, the surrounding SNPs at approximately ± 400 kb distance from CNVR_322 were not associated with the traits studied (Fig. 1c, Additional file 1: Table S3) and were not in linkage disequilibrium with CNVR_322 (Additional file 1: Table S3), indicating that this CNVR could not be detected in a genome-wide association study using single-marker in SNP array.

**Table 3:** CNVR_322 with genome-wide significant associations with embryonic mortality at 30–60 D

CNVR_ID	CNVR_type	Chr	Start (bp)	End (bp)	CNVR_length (bp)	P-value	Freq	Number of animals in 791 animals
CNVR_322	loss	8	380,524	411,389	30,865	2.19E-05	0.02	35

SNPs positions are based on the UMD3.1 assembly of the bovine genome

**Fig. 2:**
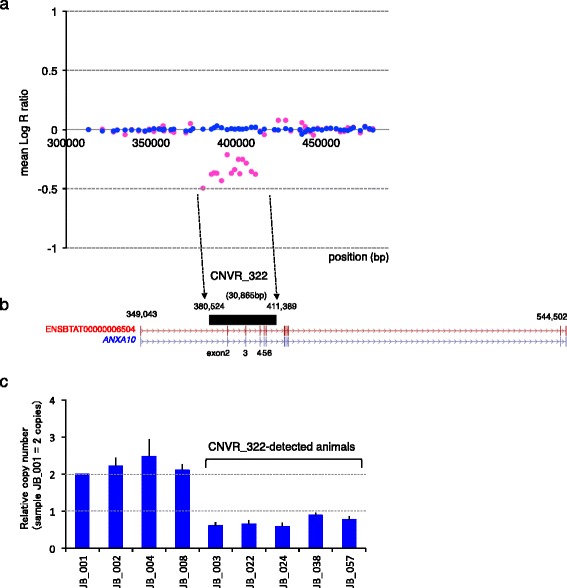
CNVR_322 overlapping with *ANXA10* gene region on chromosome 8. **a** Regional SNPs plot of CNVR_322. The mean log R ratio is indicated on the *y*-axis. The mean log R ratio of CNVR_322 animals (*magenta*) and the mean log R ratio of non-CNVR_322 animals (*blue*) were calculated from 35 animals. SNP positions were based on the UMD3.1 assembly of the bovine genome. **b** CNVR_322 (*black bar*) was visualized using the UCSC Genome Browser [54]. The Ensembl IDs and RefSeq gene symbol of *ANXA10* were labeled. **c** qPCR validation of CNVR_322. The left-most bar represents a calibrator animal (JB_001). The calibrator animal is assumed to contain two copies of the DNA segment detected from the PennCNV analysis. *Basic transcription factor 3* gene (*BTF3*), which served as an internal qPCR standard for both copies at a locus (2n), was co-amplified with the primers. The *x*-axis represents the animals. Brackets represent the CNVR_322-detected animals using the Illumina BovineHD BeadChip Array. Error bars represent the ± Standard Error of the Mean obtained from three experiments

The mean Log R ratio of 14 SNPs, which were consecutively located within a 30,865 bp window between 380,524 bp and 411,389 bp on chromosome 8, was low (Fig. 2a). We found that CNVR_322 overlapped with exons 2 to 6 of *ANXA10* (Fig. 2b). Quantitative PCR (qPCR) showed that the copy number was approximately one (Fig. 2c), which was in agreement with the expected deleted-type CNVR.

### CNVR_322 was generated from non-allelic homologous recombination by 63 bp homology arms

**Fig. 3:**
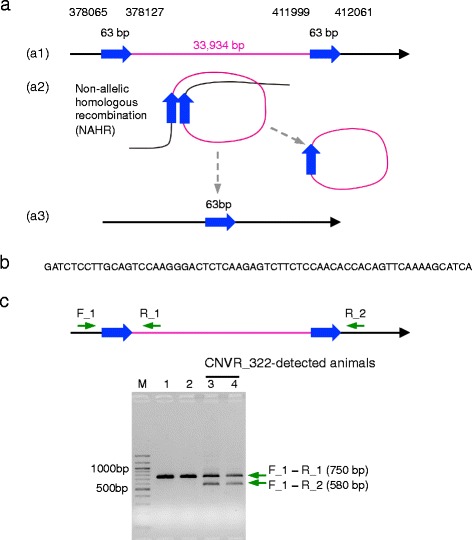
Identification of breakpoints of CNVR_322. **a** Genomic structure of CNVR_322 regions (*magenta lines*). CNVR_322 is flanked by 63 bp same-directed sequences (*blue arrows*) (a1). Analysis of sequence traces from CNVR_322-detected animals identified that 63 bp reads bridging the breakpoint were present on both side of 33,934-bp flanking regions (a1, a3). Model for generation of CNVR_322 by non-allelic homologous recombination (NAHR) (a1, a2, a3). After NAHR, the resulting region contained a 63-bp sequence (a3). The positions were based on the UMD3.1 assembly of the bovine genome. **b** Sequence of the 63-bp flanking CNVR_322. **c** Genotyping test for CNVR_322 by PCR. Schematic representation of the primer position on CNVR_322. PCR amplification within and across CNVR_322 for two homozygous wild type (lanes 1, 2) and two carrier (lanes 3, 4) animals. PCR primers F_1 and R_1 are for the wild-type allele (750 bp), and PCR primers F_1 and R_2 are for the CNVR_322 allele (580 bp). M, 100 bp ladder markers

CNVR_322 was located between 380,524 and 411,389 bp (Table 3, Additional file 1: Table S3). Thus, the breakpoints were assumed to be present between 374,545 and 380,524 bp on the centromeric side, and between 411,389 and 414,048 bp on the telomeric side (Additional file 1: Table S3). We designed a forward primer series for 374,581 to 379,470 bp, and a reverse primer series for 412,204 to 414,031 bp (Additional file 1: Table S4), resulting in a ~800-bp PCR products using CF_4 primer and TR_1 primer from the genomic DNA of CNVR_322-detected animals, but not in control animals. Sequencing showed the breakpoints, confirming 33,934 bp between 378,127 and 412,061 bp (Fig. 3a). Analysis of sequence traces from CNVR_322-detected animals identified that there were 63 bp reads bridging the breakpoint (Fig. 3a, b), were present on both sides of the 33,934 bp flanking regions in wild-type animals (blue arrows represent the 63 bp region in Fig. 3a), indicating that CNVR_322 was generated by non-allelic homologous recombination (NAHR) (see [6] for review) via the 63-bp homologous sequences (Fig. 3a). NAHR-mediated CNVs were commonly found in the genomes [22–26].

To test the genotype of CNVR_322 by PCR, we designed multiplex primers for the CNVR_322 allele (primer pair: F_1, R_2) and wild type allele (primer pair: F_1, R_1) (Fig. 3c). A 580-bp band was detected in CNVR_322-detected animals using primers F_1 and R_2 (Fig. 3c; lanes 3, 4), whereas the band was not detected in non-CNVR_322-detected animals (Fig. 3c; lanes 1, 2). Therefore, the PCR-based DNA test was used to detect the CNVR_322 allele.

### CNVR_322 creates a *ANXA10* null allele

**Fig. 4:**
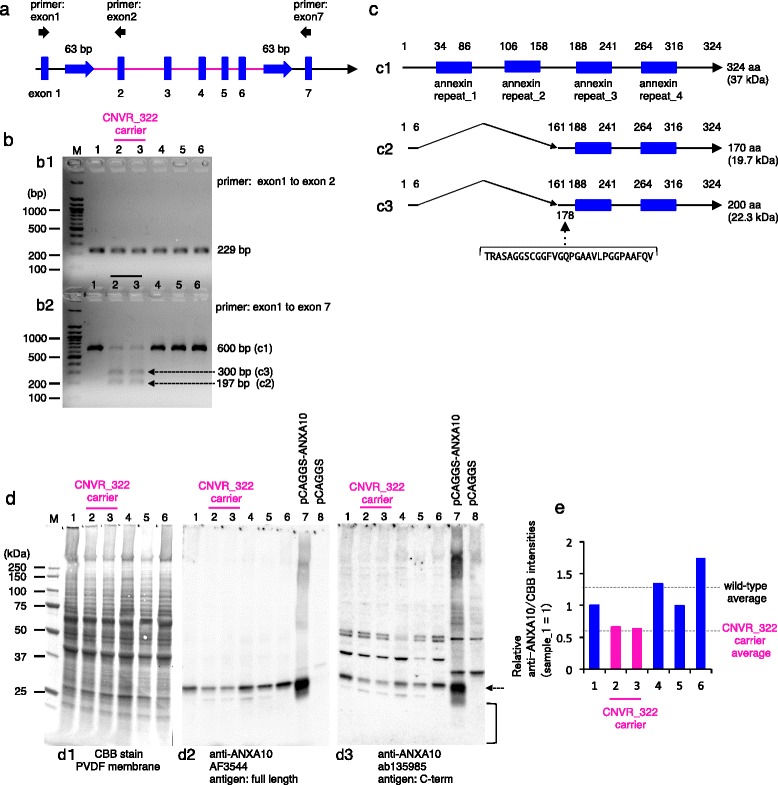
The CNVR_322 creates a *ANXA10* null allele. **a** Schematic representation of exons 1 to 7 of *ANXA10* (*blue vertical bars*) and the CNVR_322 region (*magenta line*). Primers for exons 1, 2, and 7 are represented by arrows. **b** PCR amplification of cDNA from the abomasum within and across the CNVR_322 region for four wild-type (lanes 1, 4, 5, 6) and two carrier (lanes 2, 3) animals. PCR primers for exons 1 and 2 amplified an allele (229 bp) in all animals (b1), and PCR primers for exons 1 and 7 amplified a wild-type allele (600 bp) and two mutant alleles (300 bp and 197 bp) (b2). M, 100 bp ladder markers. **c** The predicted amino acid (aa) length and molecular mass of wild-type (c1), exon 2 to 6 deleted-type (c2), and exon 2 to 6 deleted-/inserted-type (c3) is 324 aa (37 kDa), 170 aa (19.7 kDa), and 200 aa (22.3 kDa). In the exon 2 to 6 deleted-/inserted-type protein, 30 aa (*bracket*) was inserted in the 178 aa position (c3). **d** Western blotting for wild-type (lanes 1, 4, 5, 6) and two carrier (lanes 2, 3) animals using antibodies to full length (AF3544) and to the C terminal region (ab135985) of the ANXA10 protein. Recombinant bovine ANXA10 proteins were expressed in HeLa cells by pCAGGS-*ANXA10* expression plasmid and used as a positive control.　A specific band was detected for ANXA10 at approximately 30 kDa (*arrow*). Brackets represent migration areas below 30 kDa. **e** Quantitation of ANXA10 bands (d2) were relative to a wild-type animal (lane 1). Relative ANXA10 band intensities were measured using the ImageQuant TL Analysis Toolbox. The amounts of loaded proteins were calibrated using Coomassie Brilliant Blue-stained bands (d1) between 25 and 50 kDa. The dashed line represents average intensities from wild-type (*black*) and CNVR_322 animals (*magenta*)

The deletion of 34 kb, which contained exons 2 to 6, resulted in the juxtaposition of exons 1 and 7 in the *ANXA10* transcript (Fig. 2b). To examine the effect of the 34 kb deletion on the transcripts, we designed primers for exons 1 to 2 and for exons 1 to 7 (Fig. 4a) and performed RT-PCR using total RNA of the abomasum of cattle, as *ANXA10* was expressed in the abomasum (Additional file 2). We amplified a 197-bp fragment, which is consistent with the predicted size from exon 2 to 6 deleted-type ANXA10 cDNA in CNVR_322, from the carrier but not from wild-type animals (Fig. 4b2). Unexpectedly, we also found a fragment of approximately 300 bp in the carrier animals (Fig. 4b2). Sequencing of the 197-bp RT-PCR product confirmed the juxtaposition of exons 1 and 7 in the deleted-type *ANXA10* transcripts (Fig. 4c2), indicating that the exon 2 to 6 deleted-transcript of *ANXA10* was present in carrier animals (Fig. 4c2, Additional file 3). Sequencing of the 300-bp RT-PCR product resulted in the discovery of a 90 bp fragment (30 amino acid [aa]), which corresponds to the entire intron (414,996 bp to 415,085 bp) between the 7th and 8th exon of *ANXA10*, inserted into the deleted-transcript of *ANXA10* at the 75 bp position (Additional file 3, Fig. 4c3). The new transcript did not splice the intron between the 7th and 8th exon of *ANXA10* in the CNVR_322 carrier animals, whereas the transcript was not present in wild-type animals (Fig. 4b2). We hypothesize that these transcripts were in-frame of translation and the predicted molecular masses of the deleted-type (Fig. 4c2) and the deleted-/inserted-type proteins (Fig. 4c3) were 170 aa (19.7 kDa) and 200 aa (22.3 kDa, the inserted 30 aa is indicated parenthetically), respectively (Fig. 4c).

To determine whether these deleted-type ANXA10 proteins were present in the CNVR_322 carrier animals, we performed western blotting with anti-ANXA10 antibodies to the full-length and to the C-terminal of the ANXA10 protein, a region shared among the wild-type and the deleted-type ANXA10 proteins. The antibodies detected a 30-kDa band in lysates from the abomasum and cells expressing recombinant ANXA10 protein (Fig. 4d2, d3). The intensities of the 30-kDa bands were lower in the carrier animals than in the wild-type animals (Fig. 4d, e). No specific immunoreactive bands were detected in the carrier animals below 30 kDa using the antibody against the C-terminal of the ANXA10 protein (Fig. 4d; a bracket in d3), suggesting that the deleted-type ANXA10 protein could be untranslated or unstable; thus, CNVR_322 generates null alleles of *ANXA10*.

### The association of CNVR_322 was replicated in another dataset and the frequency of CNVR_322 in the Japanese Black cattle population was surveyed

**Fig. 5:**
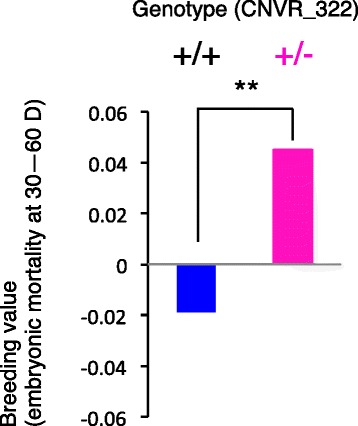
Effect of CNVR_322 on embryonic mortality at 30-60 D in 2693 Japanese Black cattle. The breeding value of embryonic mortality at 30–60 D after AI in 2552 non-CNVR_322 (+/+) animals and 141 CNVR_322 heterozygote (+/−) animals. The CNVR_322 heterozygote (+/−) was significantly associated with embryonic mortality at 30–60 D after AI, compared to non-CNVR_322 (+/+) (*t*-test, **P < 0.001)

We genotyped 2693 cows that were randomly selected from 79,617 reproductive females. The heterozygote occurred in 141 of 2693 animals, but the homozygous for the risk-allele was not observed in the population. Results showed that the CNVR_322 heterozygotes (+/−) were significantly associated with embryonic mortality at 30–60 D after AI, compared to non-CNVR_322 (+/+) animals (Fig. 5; *t*-test, *P* < 0.001).

To determine the allele frequency in the population, we further genotyped a sample of 8755 animals: cows represented 6364 animals, which were not evaluated for embryonic mortality, and steers were 2391. The heterozygote occurred in 311 cows and 71 steers, whereas the homozygous for the risk-allele was absent from the population (Additional file 1: Table S5). The frequency of the risk-allele was 0.024 in cows and 0.015 in steers (Additional file 1: Table S5), indicating that the allele was not common in the population.

### CNVR_322 has no effects on meat traits of Japanese Black cattle population

Previous studies have reported that some disease-associated alleles are accompanied by a favorable effect on other economic traits in cattle [27–29]. Meat from Japanese Black cattle is highly valued for its abundant marbling due to considerable intramuscular fat deposition [30, 31]. Therefore, the elimination of the CNVR_322 allele was validated to ensure that it exerted adverse effects on meat quality and yield traits in this breed. We did not find a significant association between CNVR_322 and any meat traits in 1156 animals (Additional file 1: Table S6). Thus, the CNVR_322 allele can be eliminated from Japanese Black cattle without adverse effects on economically important meat traits.

### Loss of maternal *Anxa10* in mice increases embryonic mortality and the mouse model recapitulates several features of the CNVR_322 heterozygous cattle

ANNEXINs, a highly conserved gene family, have four Ca^2+^ binding sites and are Ca^2+^-dependent, reverse binding to negatively charged phospholipid-binding proteins in membranes, reviewed in [32, 33]. ANNEXINs are involved in a large number of cellular processes that are associated with membrane scaffolding, vesicle trafficking, and Ca^2+^-mediated events. They also play critical roles in the inhibition of coagulation and inflammation, reviewed in [32, 33]. ANXA10, is a member of the ANNEXIN gene family; however, it has only one Ca^2+^ binding site [34] and displays a low capacity for phospholipids that are dependent on Ca^2+^ [35]. The functions of ANXA10 in vivo, including those relevant to embryonic mortality, have not yet been evaluated.

To examine whether ANXA10 is involved in embryonic mortality, we generated an *Anxa10* null mice line using the CRISPR/Cas9 system [36, 37]. We produced pups that carried an 8-bp deletion (74–81 bp) in exon 2 of *Anxa10* (Additional file 4a, b) that was predicted to cause a frame-shift and generate terminal codons at multiple positions (Additional file 4c). We confirmed that ANXA10 was not present in *Anxa10* homozygous mice (*Anxa10* ^-/-^) using western blot and immunohistochemistry (Additional file 5a, b), indicating that *Anxa10* was a null allele.

**Fig. 6:**
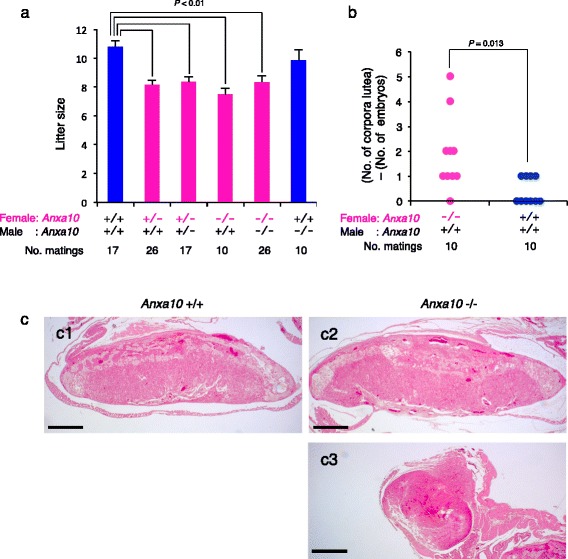
Loss of maternal *Anxa10* in mice causes embryonic mortality. **a** Litter sizes in crossbreeding experiments of *Anxa10* ^+/+^, *Anxa10* ^+/-^, and *Anxa10* ^-/-^ mice. The cross is indicated on the *x*-axis. The litter size was counted on the day of delivery. Values are represented as the mean ± SEM. **b** Difference between numbers of corpus luteum (CL) and embryos. The cross is indicated on the *x*-axis. **c** Histological analysis of the placenta at E15.5 from *Anxa10* ^+/+^ (c1) and *Anxa10* ^-/-^ female mice (c2, c3). An embryo resorption placenta is shown in c3. Scale bars: c, 1 mm

Mice heterozygous for the *Anxa10* (*Anxa10* ^+/-^) were outwardly normal, fertile, and born in appropriate Mendelian ratios. Analysis of the F2 from the mating of *Anxa10* ^+/-^ mice showed that the genotype ratios of +/+ to +/- to -/- animals were 37:76:29, indicating that embryos with *Anxa10* ^-/-^ did not exhibit embryonic mortality (chi-square test, *P* = 0.38). Crossbreeding experiments showed that litter sizes in crosses from both *Anxa10* ^-/-^ and *Anxa10* ^+/-^ females had a reduced number of pups relative to that of *Anxa10* ^+/+^ females (Tukey–Kramer post-hoc test, *P* < 0.01; Fig. 6a). In contrast, the litter sizes of *Anxa10* ^-/-^ and *Anxa10* ^+/-^ females were not statistically different (Fig. 6a), indicating that the effect of the maternal *Anxa10* allele on embryonic mortality was dominant. In cattle, although animals that were homozygous for the CNVR_322 were not observed in the population (Additional file 1: Table S5) to be directly compared with the heterozygotes, embryonic mortality was associated more with the heterozygotes than with the wild-typed animals (Fig. 5). One possibility is that the effect of the maternal CNVR_322 allele on embryonic mortality may be dominant in cattle.

In relation to this, the litter sizes in crosses from both *Anxa10* ^-/-^ and *Anxa10* ^+/-^ females were reduced by approximately 30% compared with those of *Anxa10* ^+/+^females (Fig. 6a), which indicates partial lethality. The surviving embryos had healthy placentas (Fig. 6c2) and approximately 70% pups were born normally, indicating that *Anxa10* is a quantitative trait locus for embryonic mortality in mice.

To define the stage at which embryos died, the numbers of corpora lutea (CL), implantation sites, and embryos were counted. The average number of CL at embryonic day (E) 12.5 was not different between *Anxa10* ^-/-^ female × *Anxa10* ^+/+^ male crosses and *Anxa10* ^+/+^ female × *Anxa10* ^+/+^ male crosses (Additional file 1; Table S7, *t*-test, *P* = 0.5). Consistent with this finding, the histological examination of ovaries showed that *Anxa10* ^-/-^ female mice had normal oocytes and CL (Additional file 6a), indicating that *Anxa10* ^-/-^female mice showed normal follicular development and ovulation. In addition, the average number of implantation sites at E12.5 was not different between *Anxa10* ^-/-^ female × *Anxa10* ^+/+^ male crosses and *Anxa10* ^+/+^female × *Anxa10* ^+/+^ male crosses (Additional file 1: Table S7, *t*-test, *P* = 0.9). In contrast, the numbers of CL and embryos at E12.5 were significantly different between *Anxa10* ^-/-^ female × *Anxa10* ^+/+^ male crosses and *Anxa10* ^+/+^ female × *Anxa10* ^+/+^ male crosses (Fig. 6b, *t*-test, *P* = 0.013). Consistent with these results, we also found embryo resorption at E12.5 and E15.5 (Fig. 6c3), indicating that the embryos of *Anxa10* ^-/-^females died between implantation stages E4.5 and E12.5. This stage was consistent with the stage of cattle embryo mortality at 30–60 days after AI, as cattle embryos are implanted in the uterus between 19 and 21 days after fertilization (Fig. 1a). These findings suggest that the embryonic mortality in *Anxa10* ^-/-^ female mice and in CNVR_322 heterozygote cows occurs between the implantation and the post-implantation stages and the function of maternal ANXA10 in embryo development may be shared between cattle and mice, thus, the *Anxa10* ^-/-^ mouse is a useful model for the study of embryo mortality in cattle.

ANNEXIN A5 (ANXA5), a member of the ANNEXIN gene family, is a critical factor for suppressing inappropriate blood coagulation in maternal blood vessels, reviewed in [38, 39]. Ueki et al. [40] recently reported that ANXA5 is localized in the blood vessel walls of the mouse placenta and the loss of *Anxa5* in mother mice results in the accumulation of platelet thrombosis and anemia in maternal placental blood vessels, leading to embryo death and reduced litter size in a manner similar to that of *Anxa10* ^-/-^ female mice. In contrast, we did not find platelet thrombi in the placentas of *Anxa10* ^-/-^ females, which was confirmed using antibody to an anti-integrin beta 3 (a platelet marker) (Additional file 6b). In addition, the placenta did not exhibit anemia, as both fetal erythrocytes with nuclei and maternal erythrocytes were present in the labyrinth zone of the placenta in *Anxa10* ^-/-^ females (Additional file 6c). This suggests that the mechanism underlying embryonic mortality may be different from that of the *Anxa5* ^-/-^ female mice.

*ANXA10* was abundantly expressed in the abomasum of cattle (Additional file 2) as well as in the stomach of mice (Additional file 7). The decrease in ANXA10 expression was correlated with de-differentiation and tumor progression in gastric carcinoma [41], pointing to a possible tumor suppressor role. However, the expression pattern and the de-differentiation role may not be directly connected to embryonic mortality in CNVR_322 heterozygous cows and *Anxa10* ^-/-^ female mice. Since it is presently not known how the loss of maternal ANXA10 in cattle causes embryonic mortality in the early stages of pregnancy, further investigation into the functions of maternal ANXA10 using *Anxa10* ^-/-^ mice will provide novel information regarding the underlying mechanisms of embryonic mortality in CNVR_322 heterozygous cattle.

## Conclusion

In this study, we evaluated embryonic mortality as a categorical trait with a threshold model and directly scanned the CNVs associated with embryonic mortality in Japanese Black cattle. We identified a deleted-type CNVR, which encompassed *ANXA10*, that was associated with embryonic mortality after the post-implantation stage in cattle. Consistent with this, we demonstrated that the crosses of both *Anxa10* ^-/-^ and *Anxa10* ^+/-^ female mice showed a reduced number of pups relative to that of wild-type females, and also that some embryos in *Anxa10* ^-/-^ females died between the implantation and the post-implantation stages. These results suggest that *ANXA10* is a maternal genetic factor for embryonic mortality in cattle as well as mice. As the CNVR has no effects on economically valuable meat traits in Japanese Black cattle, the elimination of the CNVR from the population will be useful in reducing embryonic mortality rates of Japanese Black cattle without adverse economic effects.

## Methods

### Collection of phenotypic data

The original data consisted of 79,617 reproductive females and 785,993 mating records from July 1993 to March 2011. Pedigree information from reproductive females was traced back two generations. Embryonic mortality was treated as a categorical trait of reproductive females. Embryonic mortality was defined by cows that received a 2nd round of AI at 18–29 days (D), 30–60 D, 61–90 D, 91–140 D, and 141 D–parturition after the 1st AI. The threshold statistical model for embryonic mortality included fixed effects of parity, breeding farm, combination of mating year and month, and age at mating as covariance:

$$ {U}_{ijkl}=\mu + PARIT{Y}_i+ FAR{M}_j+Y{M}_k+b\ast AG{E}_{ijkl}+{a}_{ijkl}+{e}_{ijkl} $$

$$ {Y}_{ijklm}=0\left({U}_{ijklm}\le t\right),{Y}_{ijklm}=1\left({U}_{ijklm}>t\right) $$

where *U*_*ijkl*_ is an unobservable underlying continuous variable (liability), μ is the overall mean, *PARITY*_*i*_ is the fixed effects of parity, *FARM* is the fixed effects of farm, *YM*_*k*_ is the fixed effects of month and year of AI, *AGE*_*ijkl*_ is age at AI of observation _*ijk*_, b is the partial regression coefficient of age at AI, *a*_*ijkl*_ is the direct additive genetic effects as random effects, *e*_*ijkl*_ is the residual effect. *Y*_*ijkl*_ is the observation _*ijkl*_ for embryonic mortality; the trait was treated as a categorical trait: if the cow was a non-recipient of a 2nd AI in the defined period, it was allocated the value 0, if the cow was a recipient of a 2nd AI in the defined period, it was allocated the value 1. The observed binary response takes the value 1 and 0 if the liability is above a fixed threshold (t) and below *t*, respectively. Genetic parameters were estimated via Gibbs sampling procedure. The THRGIBBS1F90 program [13] was used to fit a threshold animal model for embryonic mortality. This program uses a Bayesian threshold model with Gibbs sampling to evaluate the posterior density of (co)variance estimates. The Gibbs analysis was run as a single chain of 110,000 cycles. After burn-in of the first 10,000 samples, every 100^th^ sample was stored to estimate posterior means and standard deviations using the POSTGIBBSF90 program [13]. This program summarizes Gibbs samples obtained from THRGIBBS1F90 [12].

### SNP genotyping and CNVR detection

Whole blood was collected from each cow, and genomic DNA was isolated using the Easy-DNA kit (Invitrogen, Cat. #K1800-01). All sample were genotyped using the using the Illumina BovineHD BeadChip Array (Illumina, Cat. #WG-450-1002), which contains 735,293 autosomal SNPs [42], according to the manufacturer’s instructions. SNP clustering and genotype calling was performed using GenomeStudio V2011 (Illumina, version 1.9.4), and all markers passed quality control (call rate >98%). The UMD3.1 assembly was used to map SNPs position [43]. CNVR were detected as previouly reported [9], detected using PennCNV software (version June 2011) [44, 45], which incorporates factors including log R ratio (LRR), B allele frequency (BAF), marker distance, and the population frequency of B allele (PFB) into a hidden Markov model.

### Genome-wide CNV association study for embryonic mortality

Among 861 CNVRs, we selected 116 deleted-type CNVRs (Additional file 1: Table S1), which were a minor allele frequency > 0.01. The association analysis was performed on 791 samples using GEMMA software based on a linear mixed model with genomic relationships [21], which used a genetic relationship matrix estimated from SNP genotypes from the BovineHD BeadChip Array to model the correlation between the phenotypes of the sample subjects. Additionally, single-marker association analyses were performed for 791 samples using the SNPs in the BovineHD BeadChip Array.

### qPCR validation of CNVR

Real-time qPCR was performed for CNVR validation using a 7900HT Real-Time PCR system (Applied Biosystems). Primers and probes were designed for CNVR_322 (Additional file 1: Table S8). Amplification reactions (20 μl⋅well^−1^) were carried out in triplicate with 20 ng of genomic DNA, 1× Absolute QPCR ROX Mix (Thermo Scientific, Cat. #AB-1138/B), 400 nM of each primer, and 200 nM of each probe. The basic transcription factor 3 gene (*BTF3*), which served as an internal qPCR standard for both copies at a locus (2n) [46], was co-amplified with the primers (Additional file 1: Table S9). Three replicate reactions were performed for each primer pair, and a comparative C_T_ method was used to calculate the copy number [46]. ∆ C_T_ was calculated by subtracting the *BTF3* C_T_ value from the sample C_T_ value for each replicate. The average ∆ C_T_ value for the three replicates was calculated. To determine the ∆∆ C_T_, the average ∆ C_T_ of a calibrator animal, which had two copies of the DNA segment, was used. Finally, the copy number was given using the formula 2 × 2 ^-∆∆ CT^.

### Identification of the deletion breakpoint

We designed the forward primer series for 374,581 to 379,470 bp and the reverse primer series for 412,204 to 414,031 bp (Additional file 1: Table S4). The 800-bp PCR products, detected using CF_4 (377,383 to 377,402 bp) and TR_1 (412,204 to 412,223 bp), from the genomic DNA of CNVR_322-detected animals were subcloned into a pCR2.1-TOPO vector (Invitrogen, Cat. #K4500-01). Sixty-three bp on both sides of 33,934 bp flanking regions (378,065 to 378,127 bp and 411,999 to 412,061 bp were based on UMD3.1 assembly) in wild-type animals were amplified using primer pairs (Additional file 1: Table S4) and subcloned into a pCR2.1-TOPO vector. The plasmids were sequenced using M13_R and M13 (-20) primers by the BigDye Terminator v.3.1 Cycle Sequencing Kits (Applied Biosystems) and purified using CleanSEQ (Agencourt, Cat. #A29154) were sequenced, followed by electrophoresis using an ABI 3730 sequencer (Applied Biosystems).

### DNA test for CNVR_322 with multiplex PCR

Multiplex PCR was performed using F_1, R_1 and R_2 primer (Additional file 1: Table S10). The PCR conditions were as follows: initial denaturation was carried out at 98 °C for 5 min, and followed by 35 cycles, each consisting of denaturation at 98 °C for 20 s, annealing at 60 °C and extension at 72 °C for 1 min. The amplified products were separated by electrophoresis on 1.5% agarose gels with 100 bp ladder markers (NEB, Cat. #N3231L).

### Expression analysis using qPCR

For real time qPCR, we extracted total RNA from cow and mice tissues and dermal fibroblasts using RNeasy mini kits (QIAGEN, Cat. #74104) and treated it with DNase I. The cDNA was synthesized from 50 ng RNA using ReverTra Ace-α (TOYOBO, Cat. #FSK-101) with random primers, according to the manufacturer’s instructions. Bovine *ANXA10* and mouse *Anxa10* was amplified with the primers in described in Additional file 1: Table S11. Real-time PCR was performed on a 7900HT Real-Time PCR system (Applied Biosystems) using the comparative Ct method with *glyceraldehyde-3-phosphate dehydrogenase* (*GAPD*) as the internal control.

### Effect of the 34 kb deletion on *ANXA1*0 transcripts

The abomasum was collected from carrier and control steers at a slaughterhouse　(Hyogo prefecture). A portion of *ANXA10* cDNA, across exons 1 to 2 or exons 1 to 7, was amplified with primers as described in Additional file 1: Table S12. PCR products were electrophoresed by 1.5% agarose gel. The PCR product for exons 1 to exon 7 was subcloned into pCR2.1-TOPO vector (Invitrogen, Cat. #K4500-01) and sequenced using M13_R and M13 (-20) primer.

### Western blotting

To express bovine and mouse ANXA10 proteins, the coding region of bovine *ANXA10* (NM_001192058, 167–1141 bp) was amplified using PrimeSTAR Max DNA polymerase (Takara, Cat. #R045A) from cDNA of the abomasum using a forward primer (5′- GCTCTAGAatgttttgcggagactatgttcaggg -3′; uppercase letters indicate the *Xba*I linker) and a reverse primer (5′- CGGAATTCctaAGCGTAATCAGGAACGTCGTAAGGGTAgtagtcatctgcgtcacccgc -3′; uppercase letters indicate the *Eco*RI linker, and underlined letters indicate the C-terminal hemagglutinin [HA] tag for ANXA10, respectively). The PCR product was cloned into the *Xba*I and *EcoR*I sites of the pCAGGS vector [47]. The coding region of mouse *Anxa10* (NM_001136089, 166–1,140 bp) was PCR amplified from cDNA of secretroy stomach using a forward primer (5′- GCTCTAGAatgttttgcggggaatatgtccaagg -3′; uppercase letters indicate the *Xha*I linker) and a reverse primer (5′- CGGAATTCctaAGCGTAATCAGGAACGTCGTAAGGGTAgtagtcttccacatcaccagc -3′; uppercase letters indicate the *Eco*RI linker, and underlined letters indicate the C-terminal hemagglutinin [HA] tag for ANXA10, respectively). The PCR product was cloned into the *Xha*I and *Eco*RI sites of the pCAGGS vector [47]. The sequence and orientation of the insert were confirmed by sequencing. The pCAGGS Vector was used for mock transfections. For cell culture, HeLa S3 cells were maintained in Dulbecco’s modified Eagle’s medium (DMEM; Sigma, Cat. #D5796) with 10% fetal calf serum (FCS; Sigma, Cat. #F-2442) supplemented with 2 mM L-glutamine (Gibco, Cat. #25030-081) and 100 units/ml penicillin and 100 μg/ml streptomycin (Gibco, Cat. #15140-122). Using Lipofectamine 2000 (Invitrogen, Cat. #11668-019), we transfected 2 × 10^5^ cells per well in a 6-well plate with a mixture of 2 μg of the vector. The transfected cells and tissues from cattle were homogenized with RIPA buffer (25 mM Tris–HCl, pH 7.6, 150 mM NaCl, 1% NP-40 [sigma, Cat. #21-3277-2], 1% sodium deoxycholate [sigma, Cat. #D5670], 0.1% sodium dodecyl sulfate [Wako, Cat. #191-07145], protease inhibitor cocktail [Roche, Cat. #11 873 580 001], 1 mM dithiothreitol [Wako, Cat. #191-07145]) and were sonicated with Bioruptor (Cosmo bio, Cat. #UCD-250) for four cycles at a high setting, 30 s ON and 30 s OFF on ice. The expression of bovine ANXA10 was confirmed by western blotting with anti-ANXA10 antibody to full length (R&D, Cat. #AF3544, 0.5 μg/ml) and to the C-terminal of ANXA10 (abcam, Cat. #ab135985, 1 μg/ml). The expression of mouse ANXA10 was confirmed by western blotting with anti-ANXA10 antibody (R&D, Cat. #AF3544, 0.5 μg/ml and Santa Cruz, Cat. #sc-135234, 0.2 μg/ml). Immunoreactivity was detected with a horseradish peroxidase-conjugated anti-goat IgG antibody (Jackson ImmunoResearch, Cat. #205-035-108) or anti-rabbit IgG antibody (Jackson ImmunoResearch, Cat. #711-036-152) and the ECL Prime Western Blotting Detection Reagent (GE Healthcare, Cat. #RPN2232). Chemiluminescence was detected with an ImageQuant LAS 4000 (GE Healthcare) and quantified using the ImageQuant TL Analysis Toolbox.

### Replication study and frequency of the CNVR_322 in Japanese Black cattle population

For the replication study, we used 2693 cows, selected from 79,617 reproductive females that were evaluated for embryonic mortality. The 34 kb deletion was genotyped from PCR products using multiplex primers as described in Additional file 1: Table S10. The effects of the CNVR_322 were estimated as the least square means of generalized linear model (GLM) analyses. The statistical model for GLM analysis included fixed effects for the farm, birth year and CNVR_322. The genetic variance explained by the CNVR_322 was calculated based on estimates of the CNVR_322 effect and the frequency of the CNVR_322 [48]. Total genetic variance was estimated by MTDF-REML. The effect size of a CNVR_322 was estimated as the proportion of genetic variance explained by the CNVR_322.

To survey the risk-allele frequency in Japanese Black cattle, we genotyped 6364 cows that were not evaluated for embryonic mortality between 1990 and 2014 (mating records of the cows were not available) and 2391 steers from two central slaughterhouses (Tokyo Metropolitan Central Wholesale Market, Tokyo, and Nanko Wholesale Market, Osaka, Japan) that received animals from nationwide locations in Japan between 2003 and 2008.

### Association analyses between CNVR_322 and meat traits in Japanese Black cattle

Data on five carcass traits were collected from 1156 animals, which were genotyped using 50 K SNP array [49]. The 34 kb deletion was genotyped from PCR products using multiplex primers as described in Additional file 1: Table S10. Association analyses were performed using Wald test with a linear mixed model accounting for family relatedness using a genomic relationship matrix.

### Generation of *Anxa10* null mice using CRISPR/Cas9

To design a single guide (sg) RNA for *Anxa10*, we searched PAM sequences using CRISPRdirect [50] and off-target sequences using COSMID [51]. We selected 69–71 bp of PAM sequences from the start of methionine in *Anxa10* (NM_001136089, Additional file 4a) because the sgRNA-targeting site did not have off-target sequences on the genome. The forward (5′-CACCggagtgctccgcctagcatt-3′; uppercase indicates the *Bpi*I linker) and reverse primers (5′-AAACaatgctaggcggagcactcc-3′; uppercase indicates the *Bpi*I linker) were annealed and then cloned into *Bpi*I (Thermo Scientific, Cat. #ER1012)-digested pX330-U6-Chimeric_BB-CBh-hSpCas9 plasmid (pX330-sgRNA) (Addgene, Cat. #42230) [52]. The sequence of the insert and the direction were confirmed by sequencing using primer (5′-tggactatcatatgcttacc-3′). To confirm the activity of targeted endonucleases, targeted regions of *Anxa10* were cloned into pCAG-EGxxFP plasmids (Addgene, Cat. #50716) [37] to monitor the single strand annealing activity [37]. The targeted regions of *Anxa10* were amplified using PrimeSTAR Max DNA polymerase (Takara, Cat. #R045A) from genomic DNA of C57BL/6NJ mouse using a forward primer (5′-CGGGATCCtgggggtaaaaagtggtgaa-3′; uppercase indicates the *Bam*HI linker) and a reverse primer (5′-CGGAATTCcaatggtcagtgtgggtcag-3′; uppercase indicates the *Eco*RI linker). Using Lipofectamine 2000 (Invitrogen, Cat. #11668-019), we transfected 2 × 10^5^ Cos-7 cells per well in a 6-well plate with a mixture of 0.5 μg of the pX330-sgRNA and 0.5 μg of pCAG-EGxxFP to SSA activity. After 24 h transfection, cells were examined with a confocal microscope (FV1000, Olympus Optical) and EGFP-positive cells were counted using ImageJ 1.46 [53].

To inject the plasmids pronuclear, BDF1 (CLEA-Japan) female mice were superovulated using 5U/body PMSG (ASKA) and 5U/body hCG (ASKA) and mated with BDF1 males. Fertilized eggs were collected from the oviduct and cultured in KSOM medium (Millipore, Cat. # MR-020P-5 F). The pronuclear stage eggs were injected with 5 ng/μl pX330-sgRNA (10 mM Tris–HCl, pH 7.5, 0.1 mM EDTA). The eggs were cultured in KSOM overnight then transferred into the oviducts of pseudo-pregnant ICR female mice (CLEA-Japan). The tail genomic DNA were amplified by TaKaRa Ex Taq HS DNA polymerase (TaKaRa, Cat. #RR006) using forward primer (5′-tttcatggaaattcatacaatctctt-3′) and reverse primer (5′-accttggagcattgagtcat-3′). The PCR conditions were as follows: initial denaturation was carried out at 98 °C for 5 min, and followed by 35 cycles, each consisting of denaturation at 98 °C for 20 s, annealing at 60 °C for 30 s and extension at 72 °C for 1 min. The PCR product was directly sequenced using reverse primer by BigDye Terminator v.3.1 Cycle Sequencing Kits (Applied Biosystems) and purified with CleanSEQ (Agencourt, Cat. #A29154) were sequenced, followed by electrophoresis using an ABI 3730 sequencer (Applied Biosystems).

### Histological examination and immunohistochemistry

For histopathological examination, the tissue were fixed with 4% paraformaldehyde (PFA) and embedded in paraffin (JUNSEI, Cat. #58290-1501) using standard procedures. Sections (3 μm) were stained with Hematoxylin & Eosin.

For ANXA10 immunostaining, cattle abomasum samples were collected into cryotube and snap frozen with liquid nitrogen. Tissues were trimmed using cryostat blade (Leica, Cat. #14035838926) and then embedded in Tissue-Tek O.C.T. compound (Sakura, Cat. #4583) and frozen. Section (15 μm) was fixed in 4% PFA at 4 °C for 15 min. Mice stomach tissue were fixed with 4% PFA, and the tissues were rinsed with PBS, 30% sucrose/PBS and embedded in O.C.T compound. Sections (20 μm) were washed with 0.05% Triton x100 (Wako, Cat. #168-11805) in PBS. For ANXA10 immunostaining, the sections were immunostained with anti-Annxin A10 antibody (R&D, Cat. #AF3544, 5 μg/ml) in Canget signal immunostain solution A (TOYOBO, Cat. #NKB-401) for 1 h. For platelet thrombi, the sections were immunostained with anti-integrin beta 3 [EPR2417Y] (abcam, Cat. #ab75872, 1:250) [40].

## Additional files

Additional file 1: Table S1. Detailed features of deleted-type CNVRs on autosomes in this study. Positions are based on the UMD3.1 assembly of the bovine genome. ^1^ Multiple: CNVR contained more than one CNV in the same animal. **Table S2.** CNVR_322 with genome-wide associations with embryonic mortality. Embryonic mortality was defined by cows that received a second round of AI at 18–29 days (D), 30–60 D, 61–90 D, 91–140 D, and 141 D–parturition after the 1st AI. **Table S3.** Detailed features of CNVR_322 and the surrounding SNPs. Positions are based on the UMD3.1 assembly of the bovine genome. **Table S4.** Primer information for identification of CNVR_322 breakpoint. Positions are based on the UMD3.1 assembly of the bovine genome. **Table S5.** Genotype frequencies of CNVR_322 in 6364 cows and 2391 steers. **Table S6.** *P*-value of association between CNVR_322 and meat traits in 1156 Japanese Black cattle. ^1^ YE (%) = 69.419 + 0.13REA + 0.667RT - 0.025CW - 0.896SFT. **Table S7.** Number of corpora lutea and implantation sites in *Anxa10* ^-/-^ and *Anxa10* ^+/+^ female mice. **Table S8.** Primers and probes for CNVR_322. Positions are based on the UMD3.1 assembly of the bovine genome. **Table S9.** Primers and probes for basic transcriptional factor 3. Positions are based on the UMD3.1 assembly of the bovine genome. **Table S10.** Information for multiplex PCR primers for CNVR_322. Positions are based on the UMD3.1 assembly of the bovine genome. **Table S11.** Primer and probe for bovine *ANXA10* and mouse *Anxa10* expression. **Table S12.** Primer information for exons 1 to 2 and exons 1 to 7 of *ANXA10*. (XLSX 37 kb)
Additional file 2: Relative *ANXA10* expression levels in Japanese Black cattle. Tissues and cells are indicated on the *y*-axis. Total RNA was extracted from tissues (1 -16) and primary dermal fibroblasts (17) of two female Japanese Black cattle. Relative gene expression levels in the different tissues are shown as the means of the quantities relative to the value for liver (dotted line). (PDF 40 kb)
Additional file 3: cDNA sequence of bovine *ANXA10*. The boundaries between exons 1 and 2 and between exons 6 and 7 are indicated by arrows (magenta). Sequences of cDNA from CNVR_322 carrier animals were deleted exons 2 to 6 (yellow marked sequences). Sequencing of the 300-bp RT-PCR product from the CNVR_322 carrier animal shows a 90 bp fragment, which corresponds to the entire intron (414,996 bp to 415,085 bp) between the 7th and 8th exon of *ANXA10*, inserted into the deleted-transcript of *ANXA10* at the 75 bp position (bracket). (PDF 130 kb)
Additional file 4: CRISPR/Cas9 mediated *Anxa10* deletion in mice. (a) The PAM (black underline) and the 8 bp deletion (magenta underline) in base sequence (NM_001136089) and amino acid sequence of *ANXA10*. (b) The 8 bp deletion was identified at sgRNA-targeted locus (bracket, sgRNA recognition site; black underline, PAM sequence; magenta underline, the 8 bp deletion sequence). Sequence electropherogram of reverse primer represents wild-type, 8 bp deletion homozygote (*Anxa10* ^-/-^), and 8 bp deletion heterozygote (*Anxa10* ^+/-^), respectively. (c) The 8 bp deletion creates a premature stop codon at multiple positions (stop, asterisks). (PDF 288 kb)
Additional file 5: Expression of ANXA10 protein in *Anxa10* ^+/+^, *Anxa10* ^+/-^, and *Anxa10* ^-/-^ mice. (a) Western blotting for stomach from *Anxa10* ^+/+^, *Anxa10* ^+/-^, and *Anxa10* ^-/-^ female mice using antibody to full length (AF3544) and to internal region (sc-135234) of the ANXA10 protein. Recombinant mouse ANXA10 protein were expressed in HeLa cells by pCAGGS-*Anxa10* expression plasmid and used as positive control. A 30-kDa band was reduced in *Anxa10* ^+/-^ female mouse that was not detected in *Anxa10* ^-/-^ female mouse (arrow). (b) Localization of ANXA10 in the stomach from *Anxa10* ^+/+^ (b1, b2) and *Anxa10* ^-/-^ (b3, b4) female mice. The section was counterstained with DAPI (blue). ANXA10 immunoreactivities were localized at the apical membrane of mucous cells (green), which were detected only in *Anxa10* ^+/+^ (b1, b2). Scale bars: b1, 100 μm; b2 50 μm. (PDF 4572 kb)
Additional file 6: Ovaries and placentas in *Anxa10* ^+/+^ and *Anxa10* ^-/-^ female mice. (a) Histological analysis of the ovaries at E15.5 from *Anxa10* ^+/+^ (a1) and *Anxa10* ^-/-^ female mice (a2). The corpora lutea and the follicles are indicated by asterisks and arrows, respectively. (b) Sections from the placenta at E12.5 from *Anxa10* ^+/+^ (b1) and *Anxa10* ^-/-^ female mice (b2) were immunostained with anti-integrin beta 3 antibody (green) and counterstained with DAPI (blue). lab, labyrinth; jz, junctional zone; dec, decidua. (c) Histological analysis of the placenta at E15.5 from *Anxa10* ^+/+^ (c1) and *Anxa10* ^-/-^ female mice (c2). Nucleus fetal erythrocytes (arrows) and maternal erythrocytes in the labyrinth zone of placenta (c1, c2). Scale bars: a1, c2 100 μm; b1, 50 μm. (PDF 16202 kb)
Additional file 7: Relative *Anxa10* expression levels in mice. Tissues are indicated on the *y*-axis. Total RNA was extracted from tissues (1–20) of mice at 15.5 days of pregnancy, except for uterus (3) from non-pregnant mice. Relative gene expression levels in the different tissues are shown as the means of the quantities relative to the value for liver (dotted line). (PDF 42 kb)

## Abbreviations

AI: Artificial insemination
CL: Corpora lutea
CNV: Copy number variation
CNVR: CNV region
GWAS: Genome-wide association study
LD: Linkage disequilibrium
NAHR: Non-allelic homologous recombination
PCR: Polymerase chain reaction
SNP: Single nucleotide polymorphism
