# Supplementary figures and images for: Loss of maternal ANNEXIN A10 via a 34-kb deleted-type copy number variation is associated with embryonic mortality in Japanese Black cattle
Source: BMC Genomics. 2016 Nov 24;17:968. doi: 10.1186/s12864-016-3312-z (PMC5122153; doi:10.1186/s12864-016-3312-z)

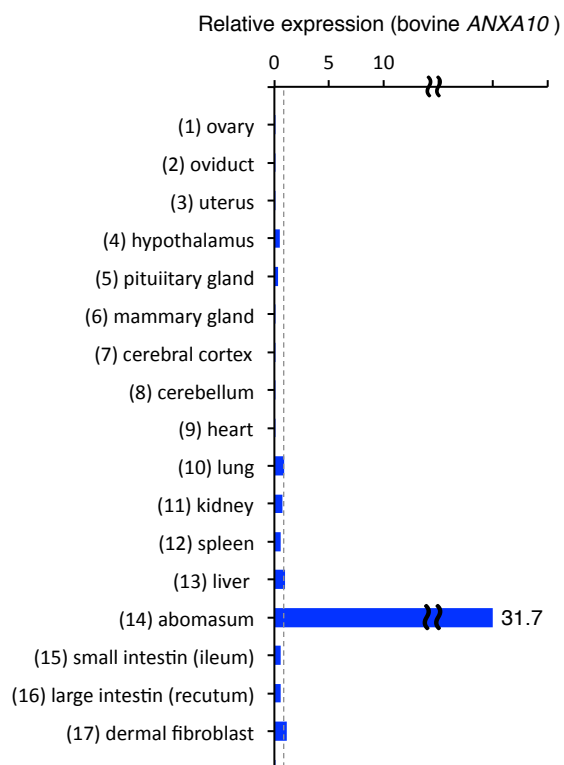

Supplement: Additional file 2: — Relative ANXA10 expression levels in Japanese Black cattle. Tissues and cells are indicated on the y-axis. Total RNA was extracted from tissues (1 -16) and primary dermal fibroblasts (17) of two female Japanese Black cattle. Relative gene expression levels in the different tissues are shown as the means of the quantities relative to the value for liver (dotted line). (PDF 40 kb) [file 12864_2016_3312_MOESM2_ESM.pdf]

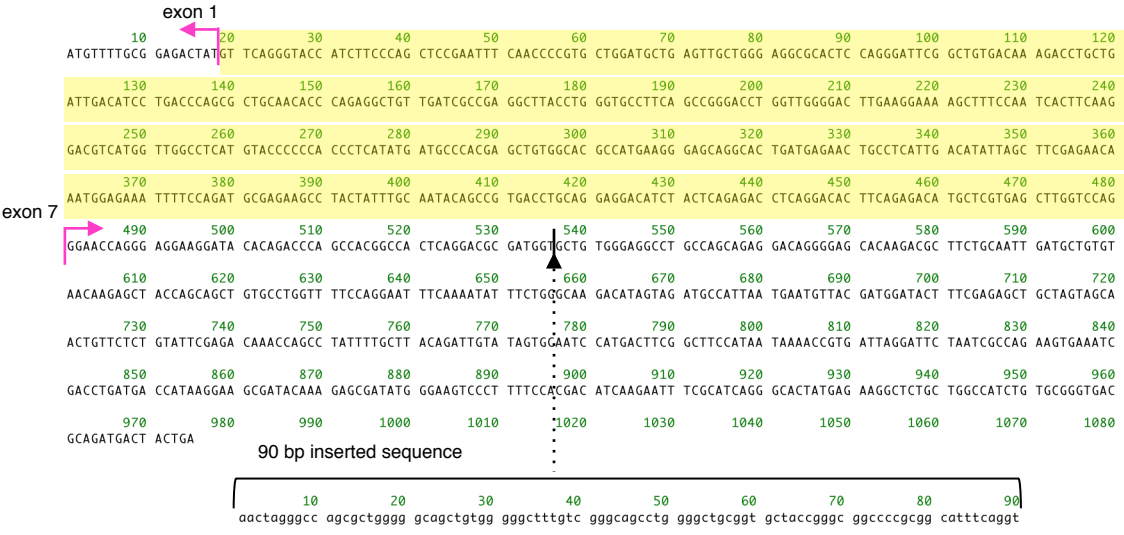

Supplement: Additional file 3: — cDNA sequence of bovine ANXA10. The boundaries between exons 1 and 2 and between exons 6 and 7 are indicated by arrows (magenta). Sequences of cDNA from CNVR_322 carrier animals were deleted exons 2 to 6 (yellow marked sequences). Sequencing of the 300-bp RT-PCR product from the CNVR_322 carrier animal shows a 90 bp fragment, which corresponds to the entire intron (414,996 bp to 415,085 bp) between the 7th and 8th exon of ANXA10, inserted into the deleted-transcript of ANXA10 at the 75 bp position (bracket). (PDF 130 kb) [file 12864_2016_3312_MOESM3_ESM.pdf]

a

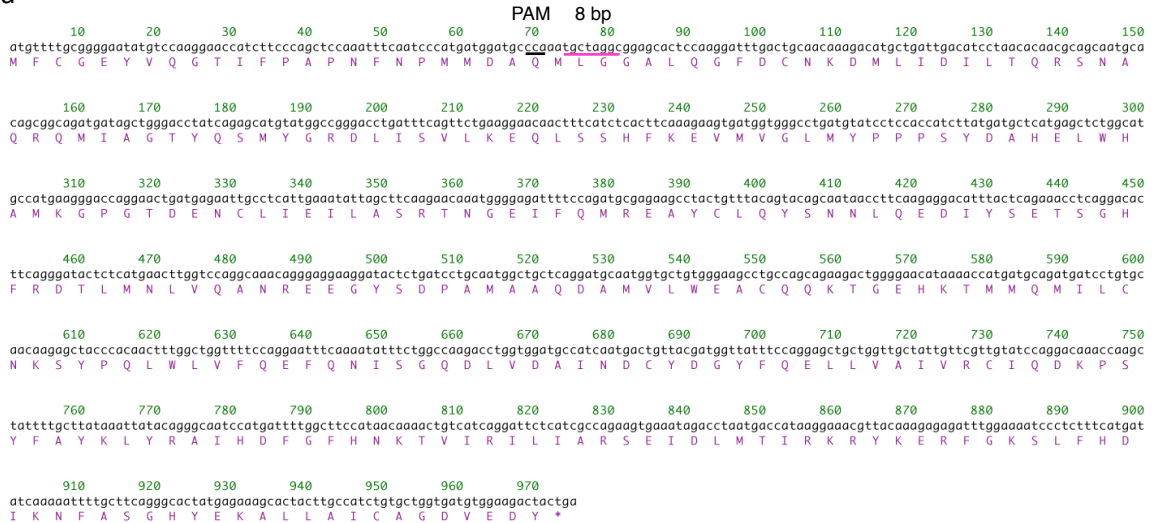

b

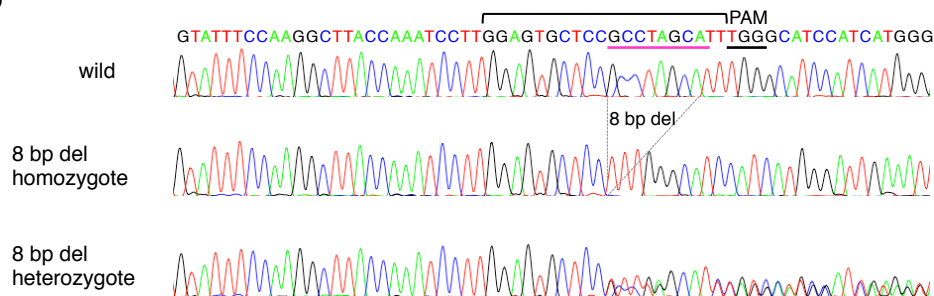

c

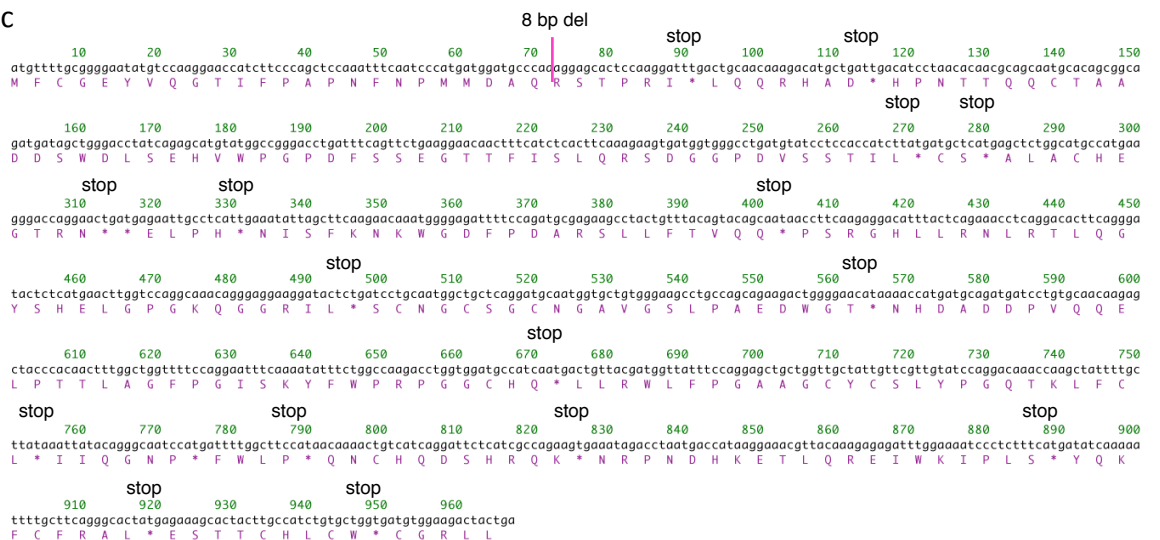

Supplement: Additional file 4: — CRISPR/Cas9 mediated Anxa10 deletion in mice. (a) The PAM (black underline) and the 8 bp deletion (magenta underline) in base sequence (NM_001136089) and amino acid sequence of ANXA10. (b) The 8 bp deletion was identified at sgRNA-targeted locus (bracket, sgRNA recognition site; black underline, PAM sequence; magenta underline, the 8 bp deletion sequence). Sequence electropherogram of reverse primer represents wild-type, 8 bp deletion homozygote (Anxa10 -/-), and 8 bp deletion heterozygote (Anxa10 +/-), respectively. (c) The 8 bp deletion creates a premature stop codon at multiple positions (stop, asterisks). (PDF 288 kb) [file 12864_2016_3312_MOESM4_ESM.pdf]

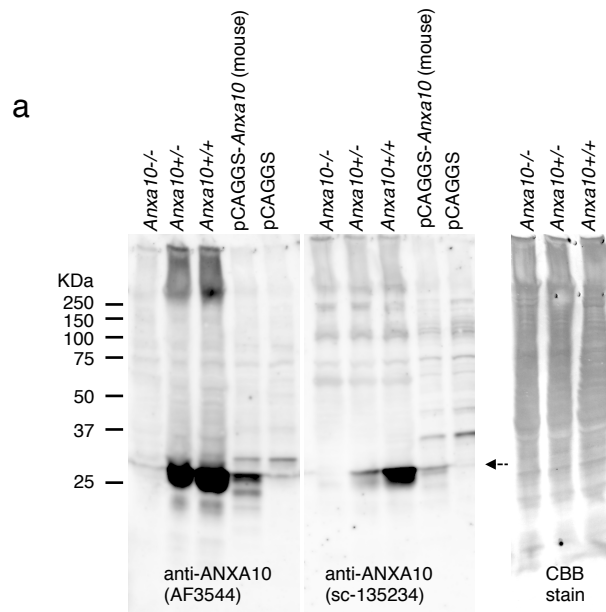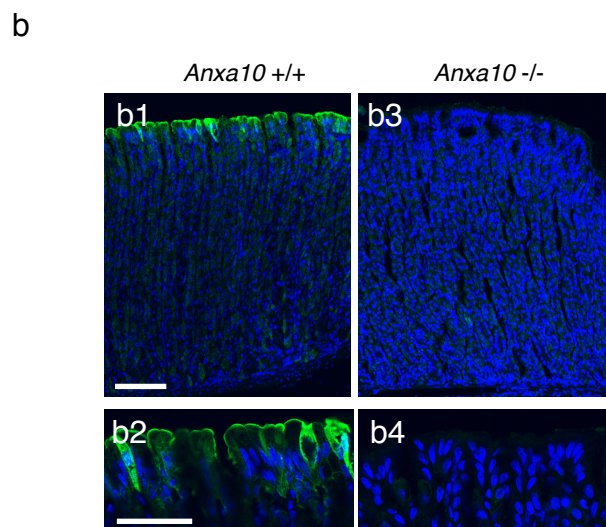

Supplement: Additional file 5: — Expression of ANXA10 protein in Anxa10 +/+, Anxa10 +/-, and Anxa10 -/- mice. (a) Western blotting for stomach from Anxa10 +/+, Anxa10 +/-, and Anxa10 -/- female mice using antibody to full length (AF3544) and to internal region (sc-135234) of the ANXA10 protein. Recombinant mouse ANXA10 protein were expressed in HeLa cells by pCAGGS-Anxa10 expression plasmid and used as positive control. A 30-kDa band was reduced in Anxa10 +/- female mouse that was not detected in Anxa10 -/- female mouse (arrow). (b) Localization of ANXA10 in the stomach from Anxa10 +/+ (b1, b2) and Anxa10 -/- (b3, b4) female mice. The section was counterstained with DAPI (blue). ANXA10 immunoreactivities were localized at the apical membrane of mucous cells (green), which were detected only in Anxa10 +/+ (b1, b2). Scale bars: b1, 100 μm; b2 50 μm. (PDF 4572 kb) [file 12864_2016_3312_MOESM5_ESM.pdf]

a

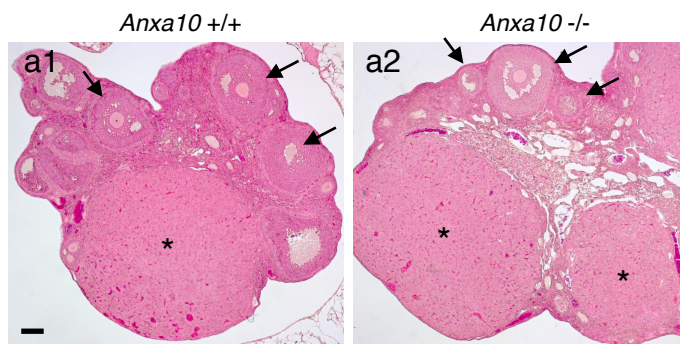

b

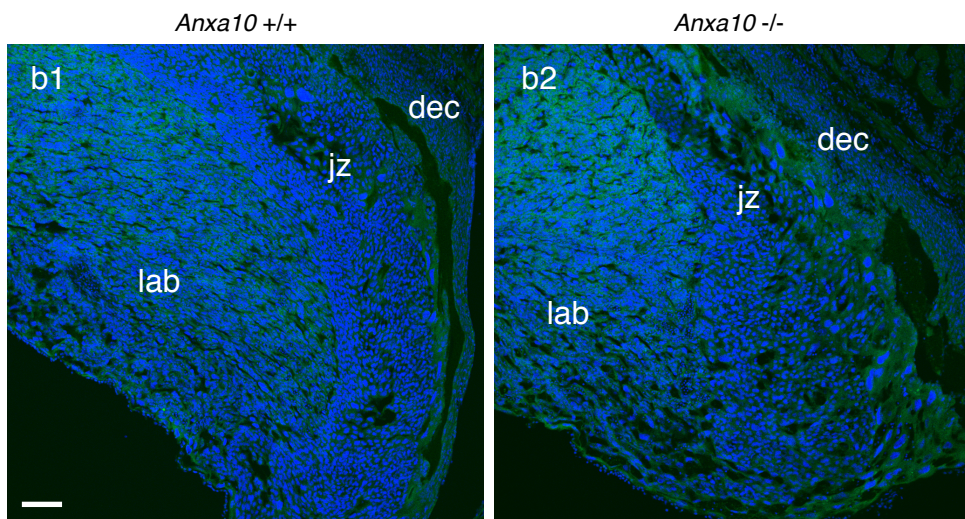

c

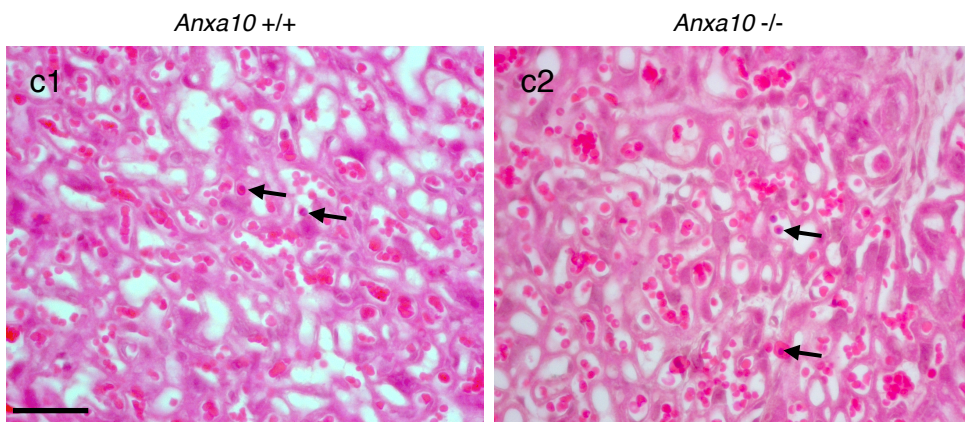

Supplement: Additional file 6: — Ovaries and placentas in Anxa10 +/+ and Anxa10 -/- female mice. (a) Histological analysis of the ovaries at E15.5 from Anxa10 +/+ (a1) and Anxa10 -/- female mice (a2). The corpora lutea and the follicles are indicated by asterisks and arrows, respectively. (b) Sections from the placenta at E12.5 from Anxa10 +/+ (b1) and Anxa10 -/- female mice (b2) were immunostained with anti-integrin beta 3 antibody (green) and counterstained with DAPI (blue). lab, labyrinth; jz, junctional zone; dec, decidua. (c) Histological analysis of the placenta at E15.5 from Anxa10 +/+ (c1) and Anxa10 -/- female mice (c2). Nucleus fetal erythrocytes (arrows) and maternal erythrocytes in the labyrinth zone of placenta (c1, c2). Scale bars: a1, c2 100 μm; b1, 50 μm. (PDF 16202 kb) [file 12864_2016_3312_MOESM6_ESM.pdf]

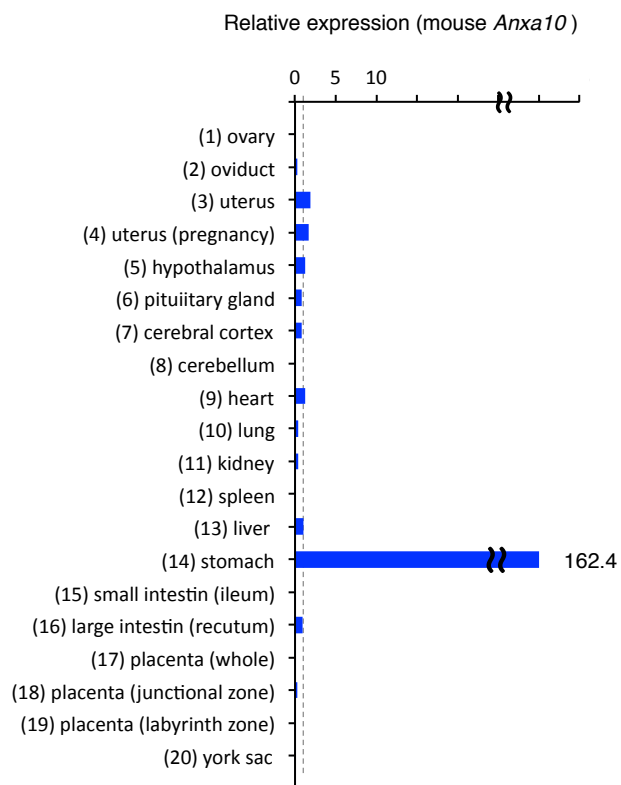

Supplement: Additional file 7: — Relative Anxa10 expression levels in mice. Tissues are indicated on the y-axis. Total RNA was extracted from tissues (1–20) of mice at 15.5 days of pregnancy, except for uterus (3) from non-pregnant mice. Relative gene expression levels in the different tissues are shown as the means of the quantities relative to the value for liver (dotted line). (PDF 42 kb) [file 12864_2016_3312_MOESM7_ESM.pdf]
